# Single-Cell Transcription Mapping of Murine and Human Mammary Organoids Responses to Female Hormones

**DOI:** 10.1007/s10911-023-09553-x

**Published:** 2024-01-30

**Authors:** Jenelys Ruiz Ortiz, Steven M. Lewis, Michael Ciccone, Deeptiman Chatterjee, Samantha Henry, Adam Siepel, Camila O. dos Santos

**Affiliations:** 1 Cold Spring Harbor Laboratory, Cold Spring Harbor, NY 11724 USA; 2 Graduate Program in Genetics, Stony Brook University, Stony Brook, NY 11794 USA

**Keywords:** Organoids, scRNA-seq, Estrogen, Pregnancy hormones, Mammary epithelial cells

## Abstract

During female adolescence and pregnancy, rising levels of hormones result in a cyclic source of signals that control the development of mammary tissue. While such alterations are well understood from a whole-gland perspective, the alterations that such hormones bring to organoid cultures derived from mammary glands have yet to be fully mapped. This is of special importance given that organoids are considered suitable systems to understand cross species breast development. Here we utilized single-cell transcriptional profiling to delineate responses of murine and human normal breast organoid systems to female hormones across evolutionary distinct species. Collectively, our study represents a molecular atlas of epithelial dynamics in response to estrogen and pregnancy hormones.

**Supplementary Information:**

The online version contains supplementary material available at 10.1007/s10911-023-09553-x.

## Introduction

Particularly during adolescence, a surge in hormones estrogen and progesterone transform the rudimentary mammary epithelium developed during embryogenesis into a complex epithelial hierarchy, marked by lineage defined cells with distinct functions [80, 95]. Yet, the most drastic postnatal developmental stage of the mammary gland occurs during pregnancy, due to the interplay of elevated levels of estrogen, progesterone and prolactin, which collectively induce the maturation of the mammary gland into a milk secretory organ [33, 79, 98, 113].

Although mouse models have been extensively used to assess the heterogeneity of the mammary gland and its associated developmental processes, organoid systems are emerging as an attractive model system that allows for the 3D culturing of mammary fragments under conditions that resemble the in vivo environment [82]. Previous studies have shown that human normal mammary-derived organoids are able to recapitulate MEC lineage diversity ex vivo [35, 97], thus representing a scalable and easily applicable model to studying the role of soluble mediators in modifying MEC function. Additionally, when grown with combinations of diverse hormone and factors, mammary-derived organoid cultures activate molecular dynamics that resemble those present during pregnancy, lactation and involution, making this system suitable to define the effects of different hormones on MECs at determined concentrations and time points [21, 115, 120]. As organoids gain traction as a model system for the study of the mammary gland in developmental biology and in cancer, a deeper characterization of this system, for mice and human cells, and the extent to which they fully recapitulate in vivo biology is needed [59].

Here, we set out to define the molecular and cellular changes induced by supplementation with estrogen, or a pregnancy-hormone cocktail, in both murine and human organoids, using single-cell RNA sequencing (scRNA-seq). We determined that murine organoid models capture the heterogeneity of intact mammary epithelial tissue, an analysis that revealed the existence of phenotypes exclusive to ex vivo cultures, marked by pathways associated with less differentiated cellular states. We also characterized the response of murine organoids to different doses of estrogen, as a way to integrate important mammary developmental and maintenance signals to ex vivo derived systems, an approach that identified molecular dynamics of hormone responsive and sensing cellular states. The single-cell characterization of mammary organoids systems grown with pregnancy-associated hormones allowed for the identification of additional cellular responses beyond those induced by estrogen. Here, the utilization of data prediction approaches allowed for the comparison of pregnancy-induced organoid states to those observed during pregnancy in mice. This analysis further illustrated the pregnancy mimicking potential of ex vivo systems, further supporting its potential as a model system to understand hormone driven mammary developmental stages.

An important advantage of utilizing 3D organoid cultures to investigate normal mammary gland development rely on the opportunity to study such process in tissues where in vivo studies are less accessible [31, 68, 84, 122]. Therefore, we further explored the robustness of mammary derived organoids, to study pregnancy-induced development of human MECs. Here we utilized organoid systems already previously described to represent the tissue cellular heterogeneity of freshly isolated breast tissue [9]. Initial characterization of these systems indicated the expression of lineage defining markers across all organoid cellular states, indicating expression infidelity induced by the culturing system. We therefore derived a set of de novo markers of cellular identities of organoid grown human MECS, by combining differentially expressed genes across untreated and pregnancy hormones treated systems, thus capturing alterations driven by signals that influence cellular behaviors.

By employing analytical cross species approaches, we also demonstrated distinct and evolutionarily conserved cellular and molecular dynamics of pregnancy hormone responses. Altogether, our efforts have generated a single-cell map of murine and human MEC-derived organoids undergoing hormone response ex vivo. This resource has the potential to pave the way for future studies exploring 3D systems to model mammary tissue development.

## Methods

### Animals

Nulliparous female C57BL/6 mice were purchased from Jackson Laboratory. All animals were housed in a 12-h light–dark cycle with controlled temperature and humidity at 72°F and 40–60%, respectively, with access to dry food and water ad libitum. All animal experiments were performed in accordance with the CSHL Institutional Animal Care and Use Committee.

### Murine Organoid Derivation and Culture

Mammary-derived organoid cultures were cultured as previously described [21], within Matrigel (Corning, CATALOG INFO) domes, submerged in Advanced DMEM/F12 +  +  + medium supplemented with 1X ITS (Insulin/Transferrin/Sodium Selenite, Gibco #41400–045) and FGF-2 at 5 nm (PeproTech, Cat# 450–33): essential media. Organoid culture medium was changed every 2 days. FGF-2 was then withdrawn from the organoid cultures for 24 h after which the treatment regimen was initiated. Organoid conditions with “low” levels of estrogen were grown with medium supplemented with 33.3 ng/mL of 17-β-Estradiol (Sigma #E2758), and those with “high” levels of estrogen were grown in the presence of 66.6 ng/mL of 17-β-Estradiol. Mouse organoid conditions to mimic pregnancy were cultured with medium supplemented with 66.6 ng/mL of 17β-Estradiol, 200 ng/mL of progesterone (Sigma #P8783) and 200 ng/mL of prolactin (Sigma #L4021). In all conditions, hormone treatment was carried out for 48 h. For the preparation of scRNAseq, organoid cultures were dissociated with 500 µL of Cell Recovery Solution (Corning® # 354253) for 30 min, followed by incubation with 500 µL of cold Tryp-LE (Thermo Fisher Scientific #12604–013) at 37 °C for 10 min. Dissociated organoids were resuspended with 1 mL medium, transferred to a 15 mL BSA pre-coated Falcon tube, and spun at 300 G for 5 min. Dissociated organoid cells were then resuspended in 1 mL of medium and submitted for library preparation and sequencing.

### Human Organoids

Established patient-derived normal breast organoid cultures [9] were cultured as previously described, within Matrigel (Corning) domes, submerged in medium containing 10% R-Spondin1 conditioned medium, 5 nmol/L Neuregulin 1 (Peprotech, 100–03), 5 ng/mL FGF7 (Peprotech, 100–19), 20 ng/mL FGF10 (Peprotech, 100–26), 5 ng/mL EGF (Peprotech, AF-100–15), 100 ng/mL Noggin (Peprotech, 120–10C), 500 nmol/L A83–01 (Tocris, 2939), 5 μmol/L Y-27632 (Abmole, Y-27632), 500 nmol/L SB202190 (Sigma, S7067), 1 × B27 supplement (Gibco, 17504–44), 1.25 mmol/L N-acetylcysteine (Sigma, A9165), 5 mmol/L nicotinamide (Sigma, N0636), and 50 μg/mL Primocin (Invitrogen, ant-pm-1) in ADF +  +  + . Organoid culture medium was changed every 3 days, and organoids were passed every 5–8 days to avoid confluency. Human MEC derived organoids were treated with pregnancy hormone concentrations same to those utilized for the growth of murine organoids (66.6 ng/mL of 17β-Estradiol, 200 ng/mL of progesterone (Sigma #P8783) and 200 ng/mL of prolactin (Sigma #L4021). We confirmed with qPCR analyses that these grow conditions induced the expression of casein genes, and utilized such analysis to define the collection time points for scRNAseq (untreated cultures, and 10 and 21 after supplementation of medium with pregnancy hormones). Cultured human organoids were processed similarly to mouse organoids prior submission for library preparation and sequencing, with organoids being dissociated with 500 µL of Cell Recovery Solution (Corning® # 354253) for 30 min, followed by incubation with 500 µL of cold Tryp-LE (Thermo Fisher Scientific #12604–013) at 37 °C for 10 min. The dissociated human organoids were likewise resuspended with 1 mL medium, transferred to a 15 mL BSA pre-coated Falcon tube, spun at 300 G for 5 min, resuspended in 1 mL of medium and submitted for library preparation and sequencing.

### qPCR Analysis

Organoid MECs were homogenized in Trizol (Thermo Fisher Scientific) for RNA extraction. Double stranded cDNA was synthesized from purified total RNA using SuperScript III Reverse Transcriptase (Thermo Scientific). QuantStudio 6 real time PCR system, Software v1.3 (Thermo Fisher) and quantification results were analyzed using the delta delta CT method. The relative mRNA expression of the target gene was determined using the ΔΔCt method and normalized against β-ACTIN mRNA levels. Comparing Casein 2 (*CSN2*) and casein 3 (*CSN3*) expression at 0 vs 21 days of EPP treatment using the Mann Whitney test yielded a significant difference in expression for *CSN2* at 21 days (*p* value = 0.0238), while *CSN3* resulted in a non-significant difference in expression (*p* value =  > 0.9999).

**Table:** 

Name	Primer sequence
Human β-ACTIN	FWD: 5’AGA GCT ACG AGC TGC CTG AC 3’
	REV: 5’AGC ACT GTG TTG GCG TAC AG
Human *CSN2*	FWD: 5’CCC ACC CAC CAG ATC TAC C 3’
	REV: 5’ CAT CAT ATT TCC AGT CTC AGT CAA 3’
Human *CSN3*	FWD: 5’GTT GCA GTT ACT CCA CCT ACG3’
	REV: 5’AGG AGA GTG TGA AGT AGT AAT TTG G5’

### scRNAseq Library Preparation and Data Analysis

Libraries were prepared with the 10X Chromium platform for single-cell libraries. The libraries were run with 3’ chemistry single end sequencing and indexing using the Illumina NextSeq 550 high output platform. Libraries from mouse samples were aligned to the mm10 genome using CellRanger v3, and human libraries were aligned to the GRCh38-2020 genome using CellRanger v6. All further data processing and analysis was completed in the Seurat package in R version 4.0.0. Initial quality control involved removing any cells with mitochondrial RNA expression over 15%, removing clusters with high ribosomal RNA expression and removing clusters with > 5,000 and < 200 features. For batch effect correction and normalization, anchors were discovered between the datasets using the FindIntegrationAnchors() function before integrating with the IntegrateData() function. Throughout the analysis and re-clustering, repeated quality control through evaluation of clusters with a large proportion of cells expressing low features or high mitochondrial RNA content were removed. These steps ensured the removal of low-quality clusters at each stage of the processing and analysis. Uniform manifold approximation and projection (UMAP) clustering using a shared nearest neighbor graph (SNN) was performed. The resolution of each clustering step with the help of Clustree [136], and all of the analysis presented here were run with a resolution of 0.3, with the exception to data analysis shown on Fig. 3F, which due to the large number of samples, was performed with a resolution of 0.2. Differences in cell numbers between datasets were analyzed with the Propeller package, which uses a robust and flexible method that leverages biological replication to find statistically significant differences in cell type proportions between groups [90]. Pseudotime estimation for murine and human culturing conditions was performed using SlingShot version 2.8.0 in R [116]. For pseudotime analyses, each colored line represents a path for one estimated lineage, which are in turn calculated based on transcriptomic similarities. The coloring of the clusters represents their position in an average path for all estimated lineages. Regulon analysis for each culturing condition and species was performed using SCENIC version 1.2.0 in R [1].

Identity assignment of epithelial cell clusters was performed using module scores based on known lineage markers [42] (Table 1), and/or top differentially expressed genes, assigned to each cluster in each Seurat object. To evaluate differentially expressed genes (DEGs) within our data, we utilized the FindMarkers() function, which completes a Wilcoxon rank-sum test to identify DEGs between clusters. For visualizing DEGs and particular genes of interest within the data, we utilized the following functions: DotPlot(), FeaturePlot(), VlnPlot() and HeatMap(). For a dendrogram analysis of the relative relatedness of the clusters, we utilized the BuildClusterTree() function using default parameters. Ternary plots were generated from resulting module scores for broad MEC lineage markers using the ternary plot() function from the vcd package. The naming of cell types is in accordance with Human Breast Cell Atlas (HBCA) discussions.

**Table 1:** Gene markers utilized to define MEC lineages

Mouse MEC lineage	Gene markers
Luminal Hormone Sensing (LHS)	*Epcam, Krt8, Krt18, Prlr, Prrg2, Ak3, Cdk19, Fxyd2, Areg, Stc2, Prom1, Esr1, Pgr, Cdo1, Gstm2, Wnt5, Cxcl15, Ly6a, Tspan9, Gltp, Cd14, Ppme1, Adck5, Dusp4, Tph1, Notch3, Itpripl1 Calca, Armcx2, Cited1, Rcan1, Pak6, Pir, Fgb, Fam83g, Il6ra, Itpripl2, Ptbp2*
Luminal Adaptive Secretory Precursor (LASP)	*Epcam, Krt8, Krt18, Col9a1, Il1rn, Itga2, Csn1s1, Car2, Csn2, Bptf, Lalba, Kit, Armcx2, Trf, Cxcl1, Ndst1, Ezh2, Ap1g1, Areg, Spp, Sfxn3, Cd14, Snx27, Mfsd5, S100a8, Lbp,, Gjb2, Notch3, Il6ra, Kctd20, Erf, Ptbp2, Ireb2, Csn3, Aldh1a3, Ceacam1, Csf3, Bmpr2, Egln3, Il1m, Lgals1, Stmn1, Tgfb3, Mki67, Lockd, H2afz, Ube2c, Prrg2, Ak3,, Cdk19, Mdk,, Ly6a, Krt14, Top2, Tagln, Cenpa, Fam83g, Rangrf, Ppme1, Hmgb2, Itpripl2, Setd7, Cwc22, Parp1, Sms, Sp110, Cenpa, Fam83g, Rangrf, Ppme1, Hmgb2, Itpripl2, Setd7, Cwc22, Parp1, Sms, Sp110, Cxcr4*
Basal Myoepithelial (BMyo)	*Epcam, Lgals1, Bptf, Krt17, Ppic, Mdk, Krt14, Krt5, Acta2, Mgp, Lmod, Lhfp, Cxcl14, Serpina3n, Cnn1, Vcam1, Nrg1, Col7a1, NexnIl17b, Mylk, Sparc, Lgr5, Jag1, Scn7a, Trp63, Lbp, Tagln, Bmpr2, Fgf1, Lipg, ArcId4, Mme, Mmp2, Igfbp3, Oxtr*

**Fig. 1:**
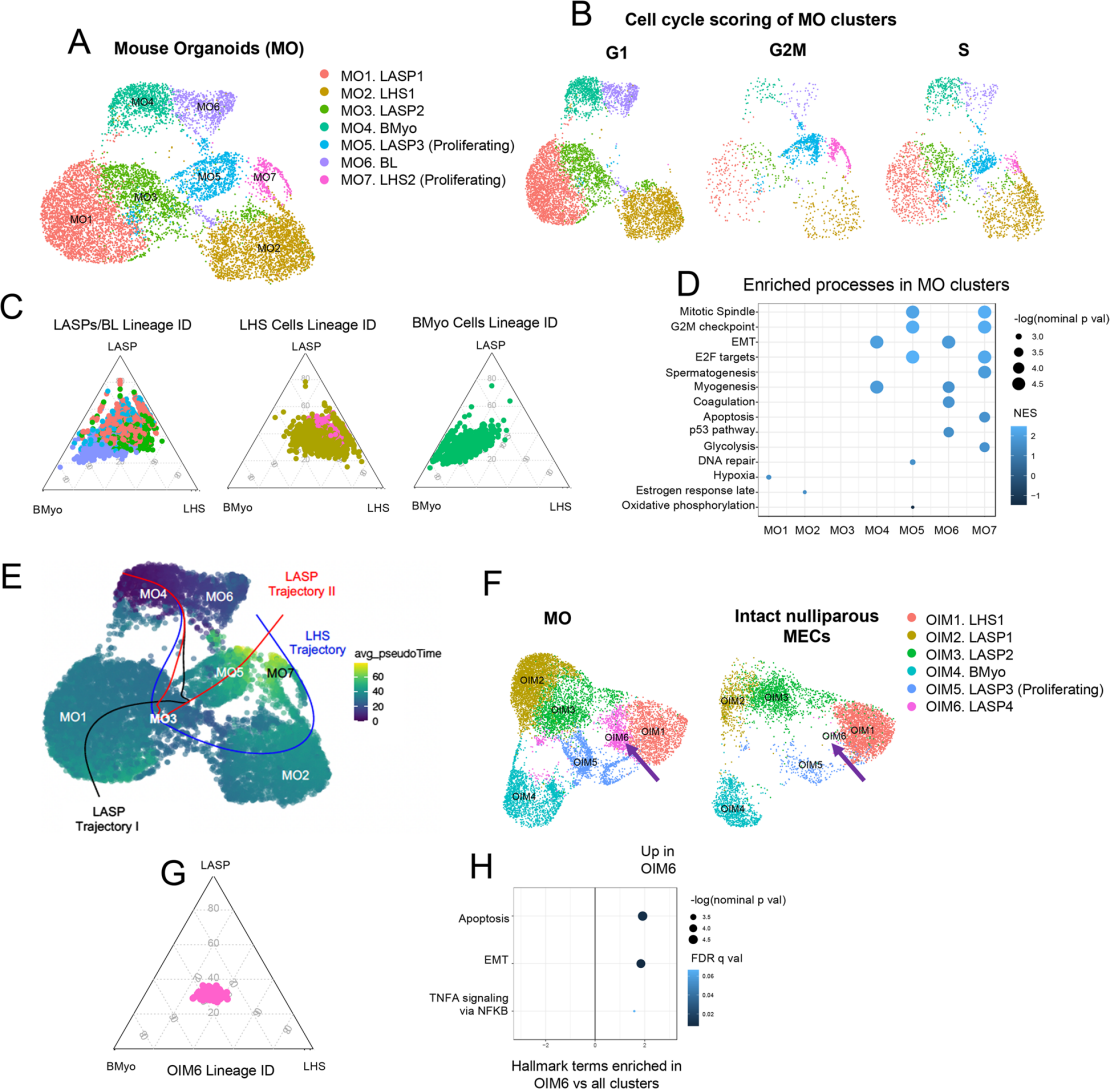
Analysis of mouse organoid MECs scRNA-seq data. **A** Mouse Organoid (MO) clusters and their given identities according to gene expression from previously described MEC markers. **B** Cell cycle scoring of MO clusters. **C** Ternary plots showing how each MO cluster scores for general lineage markers (Table S2). MO clusters are organized based on their dendrogram relationships. **D** Summary of enriched hallmark terms in each MO cluster, ordered based on each –log(nom *p*-val) for each term. Only terms with nom *p*-val < 0.05 were kept for this analysis. The color of each dot represents the NES value for each term. **E** Pseudotime analysis of MO clusters. Each line represents a lineage trajectory, and are labeled according to their terminal states. **F** Organoids integrated with intact MECs (OIM) clusters split by condition (cells originating from organoids or from intact tissue). The purple arrow is highlighting OIM6, a cluster of luminal progenitors that appears to be enriched in organoid cultures. **G** Ternary plot showing how OIM6 cells score for general lineage markers (Table S2). **H** GSEA for hallmark terms enriched in cluster OIM6. Hallmark terms are ordered based on the –log of nominal *p*-values for each term. Only terms with a nominal *p*-value (nom *p*-val) < 0.05 were kept for this analysis, in order to only show significantly enriched terms. The dots are colored based on their false discovery rate (FDR q-value), and the x-axis represents normalized enrichment scores (NES)

For data presented on Fig. 1A, organoids derived from MECs of 3 never pregnant, nulliparous female mice were utilized on the generation of scRNA-seq libraries, using the 10X Chromium platform, yielding a total 10,508 Mouse Organoid (MO) cells. For data presented on Fig. 1F, only epithelial cells (*Epcam* + , *Krt5* + , *Krt14* + , *Krt8* + , and *Krt18* +) were selected from publicly available, intact mammary tissue scRNAseq datasets, resulting in 1,986 cells originating from the Henry et al. data set and 4,025 from Bach et al. [4, 42]. After integration with mammary organoids scRNAseq (Fig. 1F), a total of 6 Organoid-MECs Integrated with Mouse-MECs (OIM) clusters, composed of 10,502 cells from organoid cultures and 6,011 cells from intact mammary tissue. An initial batch effect correction was performed for the merging of the Henry et al. data set, both Bach et al. samples and our organoid data set to account for the different number of cells in both organoids and intact tissue samples and any technical variability.

**Fig. 2:**
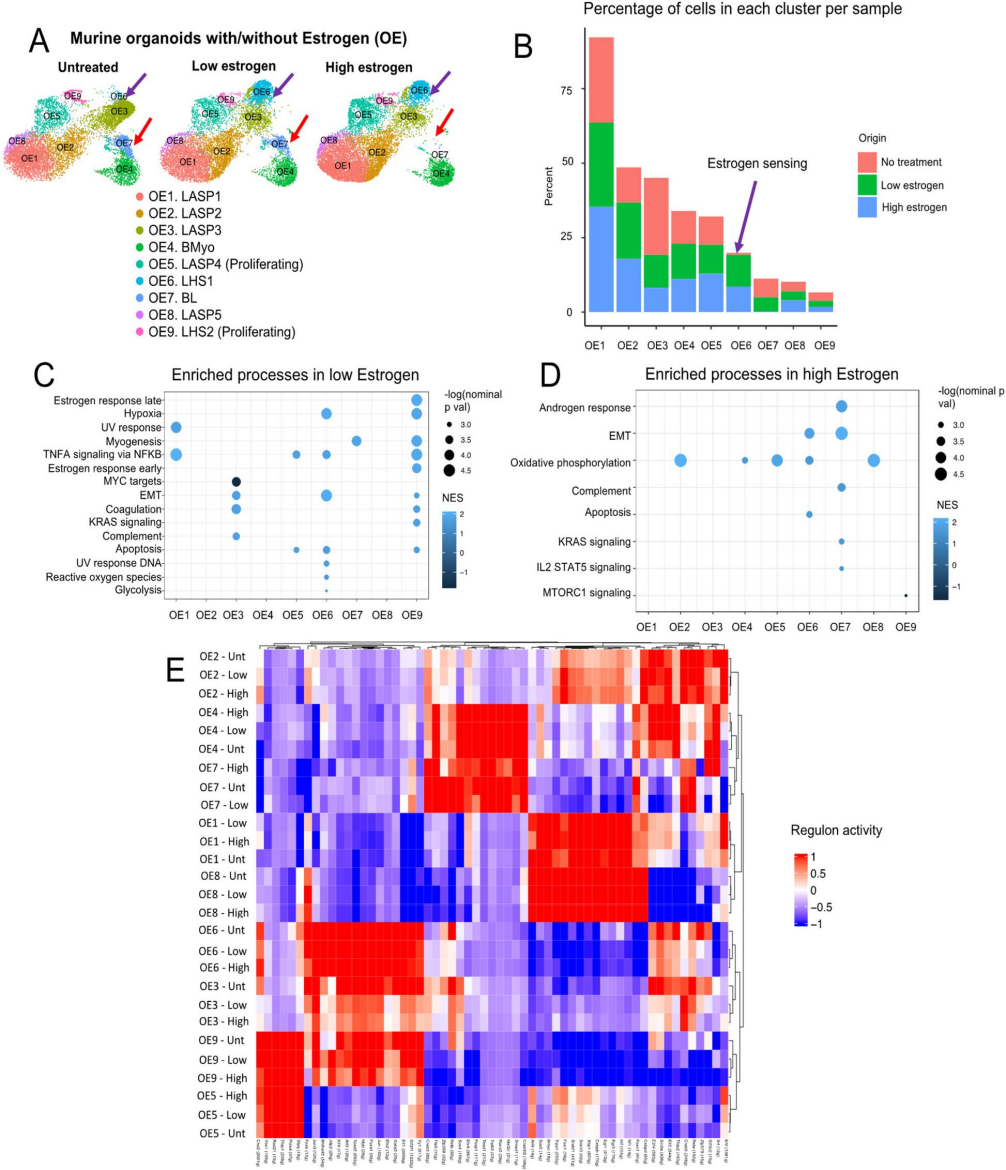
Analysis of scRNA-seq data from mouse organoid MECs with Estrogen treatment. **A** Organoids with/without estrogen (OE) clusters split by condition, highlighting Estrogen-specific cluster OE6 (purple arrow) and OE7 (red arrow), which is depleted only at a high Estrogen dose. **B** Bar plot showing percentage of cells per condition in each cellular cluster. The purple arrow highlights OE6, an Estrogen-exclusive cellular cluster. **C**, **D** GSEA for hallmark terms differentially enriched in each Estrogen treatment condition across clusters. Terms were ordered decreasingly based on their–log(nom *p*-value). Only terms with nom *p*-val < 0.05 were kept for these analyses. The color of each dot represents the NES for each term. **E** Regulon analysis showing the activities of regulons with the highest RSS per cluster and condition. The activities of each regulon are scaled to represent significant activity in one cluster or condition (red), and no significant activity (blue)

For data presented on Fig. 2, organoid cultures treated with estrogen concentrations for 48 h were prepared for scRNA-seq with the 10X Chromium platform. Quality control filtering steps and clustering alongside the untreated murine MEC-derived organoids, yielded a total of 9 clusters containing 31,802 Organoid-MECs with estrogen (OE). From these, 10,508 cells were from untreated samples, 9,695 cells were from samples treated with a low dose of estrogen (33.3 ng/mL), and 11,599 cells were from samples treated with a high dose of estrogen (66.6 ng/mL). Each of the cell cluster identities were determined once more using previously described lineage commitment markers in intact mammary tissue [42].

**Fig. 3:**
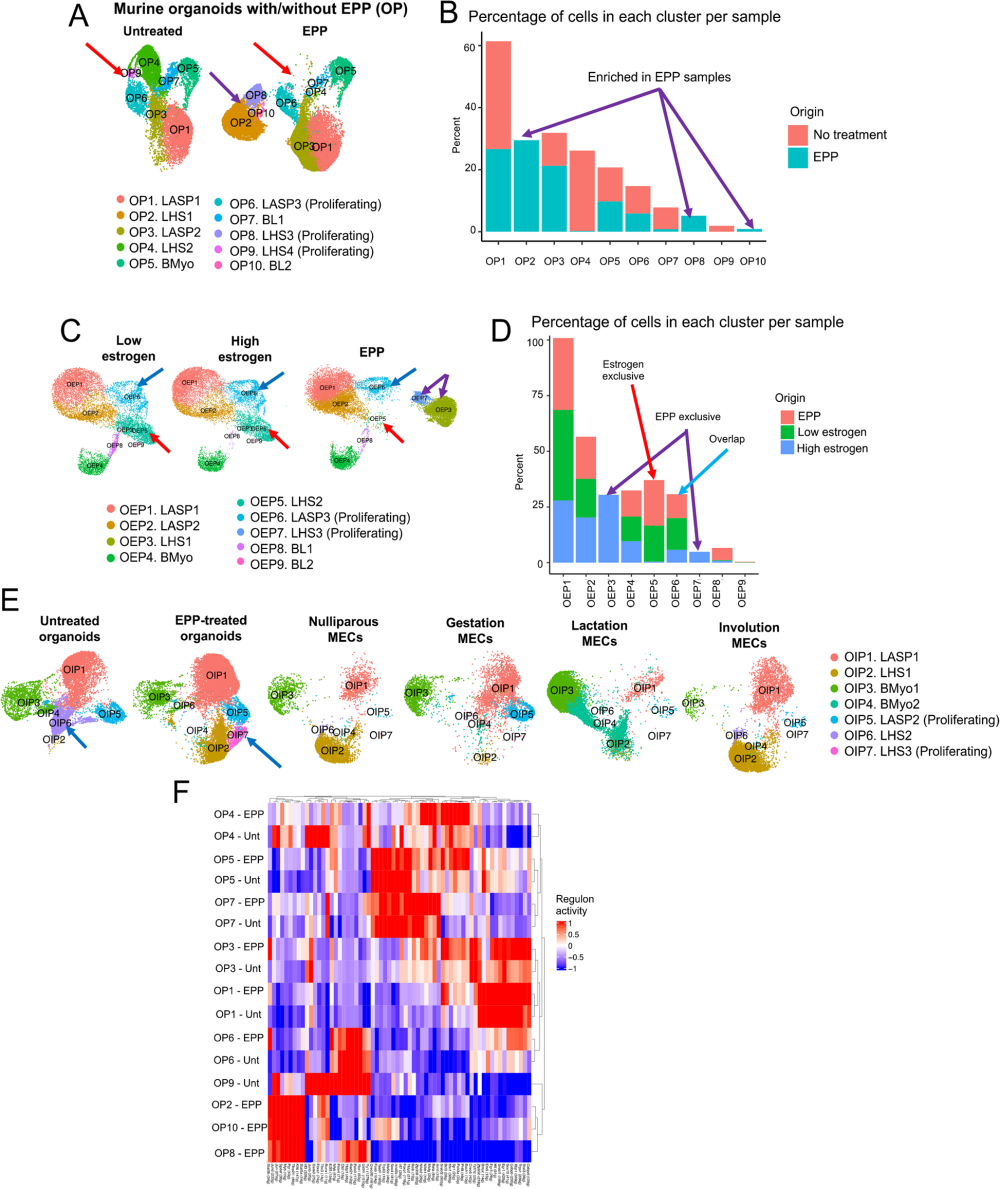
Analysis of scRNA-seq data from murine organoid MECs with EPP treatment. **A** Organoids with/without pregnancy hormones (OP) clusters split by treatment condition (untreated or EPP treatment). The purple arrow highlights EPP-enriched cellular clusters OP2, OP8 and OP10. The red arrow highlights cellular clusters depleted with EPP treatment, clusters OP4, OP7 and OP9. **B** Bar plot showing percentage of cells per condition in each OP cluster. The purple arrows highlight clusters enriched in EPP samples. **C** Analysis of scRNA-seq data from organoids treated with Estrogen or pregnancy hormones (OEP). The purple arrows highlight EPP-exclusive clusters OEP3 and OEP7, red arrows highlight estrogen-exclusive cluster OEP5 and blue arrows highlight overlapping LHS cluster between EPP and Estrogen (OEP6). **D** Bar plot showing percentage of cells per condition in each cellular cluster. **E** OIP clusters split by condition, highlighting a cellular state enriched in EPP-treated organoids (blue arrow). **F** Regulon analysis of clusters expanded in EPP

For data presented on Fig. 3, quality control steps and clustering of datasets from organoids without treatment, and those treated with estrogen, progesterone and prolactin (EPP), resulted in 10 Organoids with/without EPP (OP) clusters, with a total of 26,971 cells, 10,508 from our untreated samples and 16,463 from our samples treated with EPP. Untreated organoids and those treated with EPP were also merged with publicly available datasets from murine mammary tissue collected at different pregnancy stages [4]. After QC filtering, we obtained a total of 7 organoids integrated with MECs from a pregnancy (OIP) clusters, with a total of 4,004 cells from nulliparous (NP) MECs, 5,216 MECs from mice during gestation, 8,222 from mice during lactation, 5,607 from mice during involution, 10,497 untreated organoid cells, and 16,449 EPP-treated organoid cells.

**Fig. 4:**
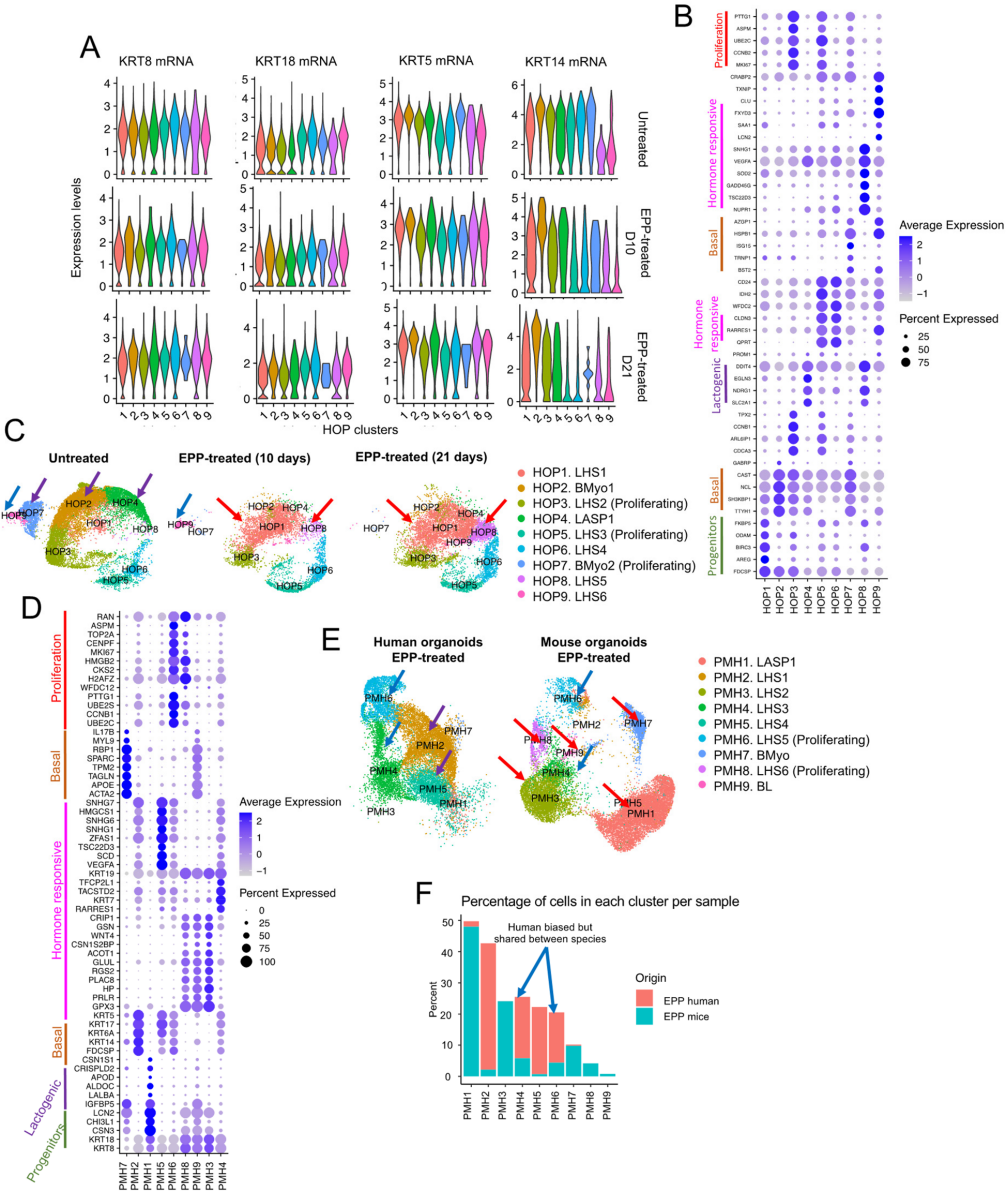
Analysis of scRNA-seq data from human organoid MECs treated with EPP and evolutionary comparisons with murine organoids. **A** Violin Plots showing the expression of cytokeratins used to classify luminal and basal populations within each human organoids with/without pregnancy hormones (HOP) cluster, divided by condition (no EPP treatment, early EPP treatment and late EPP treatment. **B** Dotplot for top DEGs per HOP cluster. Clusters are organized based on dendrogram relationships. **C** HOP clusters split by condition. The purple arrows highlight clusters enriched in organoids without treatment, and red arrows highlight clusters enriched with EPP, independent of the amount of time with EPP treatment. **D** Dotplot for top DEGs per mouse and human treated with pregnancy hormones (PMH) cluster. Clusters are organized based on dendrogram relationships. **E** PMH clusters split by species of origin. The purple arrows highlight clusters enriched in human samples, red arrows highlight clusters enriched in mouse samples, and blue arrows highlight clusters enriched in both species. **F** Bar plot showing percentage of cells per condition in each PMH cluster. Clusters enriched in both humans and mice are highlighted by the blue arrow

For data presented on Fig. 4, scRNAseq profiles of untreated human organoids, and pregnancy hormone treated ones (10 days and 21 days of EPP treatment), low quality cells were removed, yielding a total of 14,621 cells from organoids without treatment, 5,888 cells from organoids at 10 days of EPP treatment, and 8,167 cells from organoids at 21 days of EPP treatment, respectively, which were utilized on further analysis.

For data investigating similarities across species, murine genes were converted into their human orthologs before scRNAseq data integration [139]. This approach yielded a total of 7 clusters for our untreated human and murine organoid (UHM) comparison, with 14,621 cells from humans and 10,508 cells from murine organoids. Similarly, for our murine and human organoids with pregnancy hormones (PMH) comparison, the aforementioned approach yielded 5 clusters, with 14,055 cells from humans and 16,463 cells from murine MEC organoids.

Pathway analysis was performed using Gene Set Enrichment Analysis (GSEA) v3.0 and with the Molecular Signatures Database (MSigDB) Hallmark Terms [62, 77, 118]. This database was selected with the purpose of obtaining an overview of the processes each cellular cluster was undergoing. The resulting hallmark terms were further filtered based on their nominal (nom) *p*-value (< 0.05), with the purpose of only showing significant terms per cluster and/or condition. The -log(nom *p*-value) for each hallmark term was calculated so that these could be visualized based on significance. On Fig. 1, given that most clusters had similar signature gene modules, differentially expressed pathways with an adjusted *p*-value of < 0.06 were kept for further analysis.

## Results

### Determining the Cellular Landscape of Murine Mammary Organoids

#### Cell Identities of Murine Organoid Clusters

Previous studies have demonstrated the capacity of human MEC-derived organoids in retaining in vivo lineages [35, 97]. Further studies have demonstrated the feasibility of murine organoid systems to recapitulate parity-associated phenotype, such as expressing milk associated proteins and parity-associated epigenomic signatures [21, 120]. However, the heterogeneity of mammary organoids cultures, and how it recapitulates the heterogeneity of intact tissue remains to be elucidated. In order to assess the cellular and molecular heterogeneity of mammary organoids, we performed single cell RNA sequencing (scRNAseq) in organoid cells that were derived from partially digested mammary epithelium fragments of nulliparous female mice, using the 10X Chromium platform.

Utilization of previously defined markers for lineage identities in intact mammary tissue [42] allowed for robust classification of Mouse Organoid cell types (referred thereafter at MO clusters). Such analysis identified 5 populations of luminal epithelial cells, marked by the expression of both cytokeratin 8 and 18 (*Krt8*/*Krt18*) markers, (MO1, MO2, MO3, MO5 and MO7), one population of basal myoepithelial (BMyo) cells defined by the expression of cytokeratin 5 and 14 (*Krt5*/*Krt14*) (MO4), and a cluster of cells expressing both luminal and myoepithelial markers (basal-luminal cells, BL, MO6) (Fig. 1A, Fig. S1A, Table S1).

Further gene expression analysis shed light into the lineage subtypes of each cellular cluster. Expression of hormone receptors such as progesterone receptor (*Pgr*), prolactin receptor (*Prlr*) and estrogen receptor a (*Esr1*) defined luminal populations of hormone sensing (LHS) cells MO2 and MO7 (Fig. 1A and S1A). Cluster MO1, MO3 and MO5 were defined to have a luminal adaptive secretory precursor fate (LASPs), given higher expression levels of genes linked to milk synthesis, such as casein 3 (*Csn3*), and lactalbumin alpha (*Lalba*) [4, 103] (Fig. 1A and S1A). Luminal cluster MO5 (LASP3) and MO7 (LHS2) were also characterized by the expression of genes associated with highly proliferative gene signature such as marker of proliferation ki-67 (*Mki67*), ubiquitin conjugating enzyme E2 C (*Ube2c*), DNA topoisomerase II alpha (*Top2a*), thus defined as proliferative cellular states (Fig. S1A). Further cell cycle scoring analysis confirmed that epithelial cells from both MO5 and MO7 clusters were predominantly at G2M and S-phase stages of cell cycle, thus supporting that several luminal subtypes assume a proliferative state in organoid cultures (Fig. 1B).

Our analysis also defined molecular states of less differentiated cell types. We found that cells from clusters MO1, MO3 and MO5 were characterized by expression of luminal progenitor genes FXYD domain-containing ion transport regulator 3 (*Fxyd3*), cluster of differentiation 14 (*Cd14*) and claudin-3 (*Cldn3*) [3, 23, 109, 129] (Fig. S1A). Cluster MO3 cells also expressed genes associated with milk synthesis WAP four-disulfide core domain protein 18 (*Wfdc18*) and mucin-15 (*Muc15*), thus supporting a secretory progenitor state [87, 107] (Fig. S1A). Moreover, the suggested lineage identities of all organoid epithelial cell types were supported by the utilization of ternary plot analysis, which suggested an intermediate/LASP lineage signature clusters MO1, MO3, MO5), a BMyo-biased identity to BL cells (MO6), while luminal LHS (MO2 and MO7) and BMyo (MO4) clusters aligned alongside their predicted lineage identities (Fig. 1C, Table S2).

To estimate whether the LASP/intermediate signature is defining less differentiated cells, we investigated the lineage trajectory of mammary organoid cells according to their general transcription similarities, using Slingshot [116]. Our analysis predicted that cluster MO3 cells (LASP2) have a transcriptional profile that branches across multiple trajectories, including those with segments towards BL and LASP1 clusters (MO1 and MO4), and those spanning cluster MO5 (LASP3 proliferating) and BL cells (MO4) (Fig. 1E, black and red lines). Our analysis also identified cluster MO3 cells to share transcriptional programs across clusters MO2 and MO7, thus suggesting trajectories of hormone sensing state commitment (Fig. 1E, blue line). Interestingly, and independently of the shared transcriptional programs with LASP cells, BL cluster MO4 bear a pseudo-time trajectory signal closely related to BMyo cells, thus supporting its dual basal-luminal cellular state (Fig. 1E, dark blue/purple signal).

#### Signaling Pathways Enriched in Murine Organoids

We next investigated which molecular signatures were enriched in each cluster. While clusters MO5 and MO7 were enriched with pathways associated with cell division, cells from cluster MO2 were marked by processes associated with hormone sensing cells, thus supporting their above assigned cellular states (Fig. 1A, C and D, S1A). Accordingly, the BMyo state of cells from cluster MO4 were supported by the enrichment of genes associated with myogenesis and EMT-like processes [47]. Cells from LASP cluster MO1 were significantly enriched for terms involved in hypoxia. However, when considering hypoxic genes detected in our dataset, we found that most of these were involved in milk-synthesis, such as *Lalba* and *Aldoc*, supporting a LASP classification [4, 100, 103] (Table S3). BL cells (MO6) were enriched for terms similar to BMyo (MO4), as well as expressing genes involved in p53 signaling and coagulation. Notably, the genes involved in coagulation in MO6 have also implicated in EMT processes, such as fibronectin 1 (*Fbn1*) and kallikrein-related peptidase 8 (*Klk8*), supporting an potential undifferentiated state for MO6 cells [5, 46] (Table S3). Interestingly, cells from cluster MO3, classified as LASPs, did not show enrichment for specific terms in relation to all other cell types, thus suggesting an organoid cellular state that shares transcriptional signatures with all other cellular clusters. We compared MO3 to MO1, in order to explore how early LASPs (MO3) differ from those that are *Lalba* + (MO1) (Fig. S1B). The aforementioned analysis revealed that cells in MO3 are enriched for genes associated with apoptosis and EMT, both which have been associated with undifferentiated processes in mammary epithelial cells and thus suggest an increased plastic state for cells in MO3 [60].

#### Comparison of Murine Organoid and Intact MEC Transcriptional Profiles

In order to define any culture-induced changes to mammary MECs, we utilized two previously published scRNA-seq datasets from intact murine mammary tissue [4, 42] to map epithelial cell identities to our organoid data set. Integration of the epithelial portion of the intact MECs datasets and our organoid cells dataset (referred hereafter as OIM clusters) yielded 6 epithelial clusters, including those of luminal fate (OIM1, OIM2, OIM3, OIM5 and OIM6) or BMyo lineage (OIM4) (Fig. 1F, S1C, Table S1). Overall, the majority of clusters defined on intact mammary tissue are represented in organoids, with the exception of cluster OIM6 (BL) and OIM7 (LHS), which were exclusively found in organoid conditions (Fig. 1F). Interestingly, global expression hierarchical relationship across all clusters (dendrogram), indicated a closer relationship between cluster OIM1 (LHS cells) and OIM6, which lacks the expression signature of hormone-responsive cells (Fig. S1C). Conversely, OIM6 expressed elevated levels of genes in LASP cellular states such as *Csn3*, *Trf*, and *Gm42418*, suggesting an expression signature of a not fully defined luminal state (Fig. S1C). In fact, our analysis indicated that OIM6 cells are positioned in an intermediary state, right in between LHS cluster (OIM1), and LASP clusters (OIM2 and OIM3), further suggesting a transitional luminal state (Fig. 1G). GSEA for hallmark terms revealed that organoid-exclusive cluster OIM6 was significantly enriched for terms involving apoptosis and EMT, similar to what we observed in cells within MO3, thus further suggesting the presence of organoid cells with early progenitor phenotypes in culture [60] (Fig. 1H, Fig. S1B and Table S4).

Overall, our initial mapping of molecular and cellular makeup of mammary-derived organoid cultures illustrates aspects of ex vivo models that resemble intact mammary tissue, while highlighting those that are induced by several of the stimuli of a culturing system.

### Characterizing the Effects of Estrogen Treatment on Mammary-Derived Organoid Cultures

#### Cell Identities of Organoids Treated with Estrogen

Puberty represents the first key signal post-birth that drives mammary tissue expansion and MEC lineage differentiation, with increased levels of estrogen regulating cell-to-cell signaling, immune modulation, and transcription regulation [101, 124, 126]. Once developed, physiological levels of estrogen sustain mammary tissue homeostasis, with cyclical cellular dynamics throughout the estrous cycle further influencing MEC differentiation and proliferation [88]. Yet, the necessity and effects of estrogen supplementation for the growth of mammary organoid cultures has not been fully characterized.

Therefore, with the purpose of determining the effects of estrogen on gene expression, growth, and cellular heterogeneity, we set out to characterize mammary organoids treated with two concentrations of 17-β-Estradiol, 66.6 ng/mL (i.e. “high estrogen”) and 33.3 ng/mL (i.e. “low estrogen”) (referred hereafter as OE clusters) (Fig. 2A and S2A). The higher estrogen concentration aimed to replicate levels found during peak estrogen production, such as during pregnancy, while the lower concentration sought to mimic physiological levels of the hormone. Our analysis identified several clusters shared by all conditions, spanning BMyo fates (OE4), LHS states (OE6 and OE9), LASP subtypes (OE1, OE2, OE3, OE8), and BL subtypes (OE7) (Fig. 2A-B). We also identified cellular clusters marked by the expression of proliferation markers, encompassing LHS (OE9), and LASP (OE5) luminal states (Fig. 2A, S2B-C).

Further analysis of cell population distribution across organoid conditions indicated a few cellular clusters biased to specific datasets. We found a subtle decrease on the abundance of LASP3 (OE3) in organoid conditions supplemented with estrogen, perhaps suggesting that luminal progenitor differentiation in response to increased levels of estrogen can also be observed in organoid cultures [7] (Fig. 2A-B). Depletion of BL cells (OE7) was also observed in organoid cultures treated with estrogen, supporting the suggestion that estrogen supplementation may be inducing the differentiation of immature cell types, as is observed in vivo [110] (Fig. 2A-B). Interestingly, none of these cell types express hormone genes, thus suggesting a possible indirect effect of estrogen on their homeostasis/differentiation [112] (Fig. S2B). We also identified alteration to cluster of LHS cells (OE6), thus validating that expression of hormone responsive genes in subtypes of organoid cells are linked with cellular expansion in response to increased estrogen levels [30] (Fig. 2A-B, and S2B).

#### Signaling Pathways in Organoids Activated by Estrogen

We next decided to investigate global gene expression alterations across untreated and estrogen-treated organoid clusters. We first defined gene expression alterations across untreated organoids and those treated with low levels of estrogen, given that all identified clusters are represented in both conditions (Fig. 2A). Our analysis identified that clusters defined with an LHS identity (OE6 and OE9) displayed the most gene expression alterations in response to estrogen treatment, with enrichment of pathways associated with TNF-⍺ signaling via NF-κB pathways, myogenesis, EMT thus suggesting a complex net of programs that control hormone sensing states (Fig. 2C). In addition, proliferative LHS2 cells (OE9) demonstrated selective enrichment for processes associated with estrogen response (early and late) and K-Ras signaling, a pathway previously associated with estrogen receptor signaling [27] (Fig. 2C). Conversely, the population of LHS cells expanded in response to estrogen levels (OE6) was selectively enriched for pathways associated with reactive oxygen response and genes that downregulate UV responses, both potential antioxidant pathways also described to be regulated by estrogen [14, 41].

In addition to cell types defined as LHS, estrogen treatment of organoids induced alterations to specific pathways in non-hormone sensing cells. Enrichment for TNF-⍺ signaling via NF-κB pathways was observed in LASP1 (OE1), BL cells (OE7) were exclusively enriched with genes associated with myogenesis, a process that can either be suppressed or activated by estrogen levels on cellular contact dependent fashion [71, 83, 117] (Fig. 2C). We also identified the enrichment of EMT processes in LASP3 cells (OE3), an observation that may link EMT with estrogen-induced differentiation [38, 128] (Fig. 2C). Moreover, the only statistical significantly enriched pathway downregulated by estrogen was associated with c-Myc regulated processes in LASP3 cells (OE3), a signal that is essential to keep immature properties of mammary epithelial cells [91] (Fig. 2C). Interestingly, low levels of estrogen did not result in the significant enrichment of pathways in clusters of cells with BMyo fate (OE4), or certain LASP populations (OE2 and OE8), suggesting that subtypes of MECs that lack the expression of hormone genes are less affected by female hormones (Fig. 2C). Nonetheless, high levels of estrogen did enrich the aforementioned non-hormone sensing subtypes for oxidative phosphorylation-associated genes, indicating that non-hormone sensing cells are still capable of responding to hormones, at a lesser degree (Fig. 2D).

#### Regulons Coordinating Transcriptional Activities in Response to Estrogen

To assess how the regulatory networks modulating processes in each cellular sub-type might be affected by estrogen, we calculated the regulons with the highest specificity scores (RSS) for each of the OE clusters and segregated them by treatment condition (i.e., untreated, low estrogen treatment and High estrogen treatment) (Fig. 2F). Our analysis identified a series of regulons that defined overall cellular states, including proliferative (Rad21, Ybx1 and Chd2) and progenitor (Nf1, Cebpb, Sox10, Sox5, Trsp1) states [24, 27, 49, 58, 65, 75, 76, 123].

Interestingly, BMyo cells (OE4) and BL cells (OE7) shared similar transcription networks, with the exception of programs regulated by Creb3, which was also enriched in clusters defined to have luminal signatures (Fig. 2F). In fact, Creb3 has been shown to have increased activity in cells undergoing luminal-basal cellular plasticity in response to high levels of Sox9, thus supporting the suggested mixed lineage state of cells from cluster OE7 [19] (Fig. 2F). These observations suggest that mixed lineage cell types have a transcriptional identity that resembled basal states closely, with discrete alterations to luminal-biases programs.

We also identified estrogen-induced changes to transcriptional programs, encompassing both alterations to several lineage restricted programs, and those spanning several cellular states.

Analysis of BMyo cells (OE4) demonstrated a bimodal change of basal transcription programs, with the enrichment of luminal-basal plasticity regulators such as Creb3, Tfe3, and Sox4, and partial loss of programs controlled by Relb and Zfp358, both reported to be downregulated by estrogen [127, 130] (Fig. 2F). Further analysis of luminal cell types indicated enrichment of lineage specific transcriptional programs in specific cellular clusters (OE2, and OE5) (Fig. 2F). Collectively, these analyses suggest that estrogen treatment impacts the lineage programs of specific luminal and basal cell types, thus indicating cell types that are the most responsive to increased levels of female hormone.

Our investigation also identified a group of transcription programs that were altered in a lineage-independent fashion in response to estrogen levels, including programs regulated by Edr, Ehf, Creb5, Tfdp2, Elf2, Sin3a, E2f4 TFs, previously linked with regulating the cell cycle, cell growth and proliferation [40, 48, 61, 67, 92, 93] (Fig. 2F). Notably, Ehf has been reported to increase when mammary stem cells begin the process of differentiation, suggesting a role for estrogen in the maturation of hormone receptor negative MECs [133]. Collectively, our analysis indicated gains and losses of these regulon activities across all identified cellular states, thus further illustrating the complex effect of estrogen on regulatory process of all subtypes of mammary epithelial cells.

### Pregnancy Hormones Exposure, Cellular States, and Gene Expression

#### Cell Identities of Organoids Treated with Pregnancy Hormones

Mammary organoid systems have been previously optimized to mimic aspects of pregnancy-induced development of the gland, such as branching and production of milk-associated proteins, involution-like processes, and mechano-regulated actions of lactation [21, 115, 120]. Yet, it is unclear whether mimicking pregnancy-induced changes ex vivo drives cellular and transcription alterations such as those that take place in vivo. Therefore, we set out to characterize mammary organoid cultures, grown with a combination of estrogen, progesterone, and prolactin (EPP) hormones (referred hereafter as OP clusters) using scRNA-seq approaches. Our analysis identified clusters present in both untreated and EPP-supplemented conditions, encompassing cellular states of LASP fate (OP1, OP3, and OP6), BMyo lineage (OP5), in addition to lineages more abundant in untreated organoids (LHS clusters OP4, OP9, and BL cluster OP7), and those expanded in EPP-treated conditions (LHS clusters OP2, OP8, and BL cluster OP10) (Fig. 3A-B and Fig. S3A). Amongst these clusters, we identified highly proliferative cells in both conditions (OP6) (Fig. 3A-B and Fig. S3B-C).

#### Signaling Pathways in Organoids Activated by Pregnancy Hormones

We next defined the pathways differentially expressed in response to treatment with EPP. Across the cellular clusters that were present in both untreated and EPP-treated conditions, which encompassed hormone negative cell types (OP1, OP3, OP5, and OP6), we found clusters with no statistically significant enrichment for specific terms (OP1, and OP3, LASP identity), indicating cellular stages that were minimally affected by pregnancy hormone treatment (Fig. S3D). Conversely, clusters identified as BMyo lineage (OP5) and proliferating LASPs (OP6) were enriched for term that were related to their lineage specific developmental state (such as myogenesis and EMT) [71, 83, 117], or cellular state (mitotic spindle and G2M checkpoint for OP6), suggesting that similarly like estrogen alone, pregnancy hormones can induce indirect transcription changes in hormone negative cells (Fig. S3D).

We also found that clusters biased towards untreated conditions (OP4, OP7, OP9) and those more abundant in EPP-treated samples (OP2, OP8, OP10) represented very similar cellular identities, with LHS cells and BL fates, suggesting that pregnancy hormones act on cellular states present prior to hormone treatment (Fig. S3A-B and Fig. S3B). In fact, LHS clusters OP4 (untreated condition), and OP2 (EPP condition) were enriched for similar pathways, with the exception of OP4 which was also enriched for p53 signaling (Fig. S3D). Moreover, similar pathways were present in BL cells clusters OP7 (untreated condition) and OP10 (EPP condition), with the specific enrichment of p53 pathways in cells from OP7 cluster. The hormone expression on cells from OP10 cluster was linked to an enrichment of estrogen response and hypoxia, pathways also associated with pregnancy signals, thus suggesting that hormone regulated pathways are also synchronized in more immature cell types [108] (Fig. S3D). Collectively, this pathway analysis mapped the transcriptional alteration to organoid cultures in response to pregnancy hormones.

#### Defining the Individual and Collective Effects of Hormone Supplementation to Cultures

We next investigated whether the pregnancy-hormones induced changes were driven collectively by estrogen, progesterone and prolactin, or rather represent alterations regulated by estrogen alone. In doing so, we compared the transcription and cellular dynamics of estrogen treated organoids (OE clusters, Fig. 2), with those present in organoids cultured with EPP (OP clusters). This analysis yielded 9 clusters (referred thereafter as OEP clusters), encompassing populations of BMyo cells (OEP4), LASPs (OEP1, OEP2, OEP6), LHS (OEP3, OEP5, OEP7), and BL cells (OEP8, OEP9) (Fig. 3C-D and S3E). We found that that the majority of clusters are present in both culturing conditions, with the exception of 2 populations of LHS cells (clusters OEP3 and OEP7) which were exclusive to conditions treated with EPP, thus indicating cellular dynamics that only take place when estrogen, progesterone and prolactin are in place (Fig. 3C-D and S3E).

To more accurately identify estrogen induced changes, we next defined an estrogen core signature, based on the previously identified estrogen-induced cluster LHS1 cells (Fig. 2, OE6 cluster), and asked whether these markers were also present in populations of cells grown with EPP (Fig. 3A, OP clustering). We found that LHS cells states in EPP-treated samples (clusters OP2 and OP8 from Fig. 3A) and EPP-biased BL cells (clusters OP10 from Fig. 3A) showed high score expression, thus suggesting that estrogen alone induces the expansion of these cellular states (Fig. S3F).

#### Comparisons of Organoids Supplemented with Pregnancy Hormones and MECs from an Intact Pregnancy Cycle

In order to assess whether clusters identified in our hormone treated culturing system were also represented during pregnancy in mice, we performed a data integration analysis, comparing EPP -treated organoid datasets with publicly available profiles generated from MECs during gestation, lactation, and involution [4]. This approach yielded 7 clusters with varied abundance across all pregnancy-associated conditions (referred hereafter as OIP clusters) (Fig. 3E and Fig. S3G-J). We identified clusters present all datasets, including those of LASPs (OIP1), LHS (OIP2), and BMyo (OIP3) identities, suggesting cellular states that are agnostic to fluctuations of pregnancy hormones (Fig. 3E).

Further analysis identified populations of cells biased towards specific stages of pregnancy-induced development, and with some representation in organoid cultures treated with EPP. For example, we identified a population of proliferating LASPs (cluster OIP5) to be more abundant in EPP-treated organoid cultures and in mammary tissue during gestation, suggesting populations of cells that are activated by hormones early during the pregnancy cycle (Fig. 3F). BMyo cells that express oxytocin receptor (Oxtr, cluster OIP4), were found to be biased towards samples from mammary tissue during lactation, suggesting cellular states linked with responses with processes beyond milk production (Fig. 3E and S3H). Proliferative LHS3 cells (OIP7) with greater bias towards EPP-treated organoids, and LHS2 cells (OIP6) more abundant in untreated organoids, displayed limited representation across datasets generated from MECs, thus suggesting cellular states enhanced by culturing conditions (Fig. 3F).

#### Regulons Coordinating Transcriptional Activities in Response to Pregnancy Hormones

We next set out to define the pool of enrichment for regulons across all clusters, in response to pregnancy hormones (Fig. 3F, based on 3A clustering). This approach allowed for the identification of canonical hormone sensing transcription programs induced in EPP-treated conditions, such as those regulated by Stat5a and Stat5b, Pgr, Jun, Klf6, Tfcp2l1, Myb, Spdef transcription factors [2, 6, 8, 11, 20, 25, 26, 29, 64, 85, 94, 125, 135] (Fig. 3F). Our analysis also identified several regulons enriched in EPP-treated condition, in a non-lineage fashion, which included programs regulated by Tcf7l2, Phfl8, Sp1, Arntl, Nfkb1 factors in LHS cells (OP4), BMyo subtypes (OP5 and OP7), and in LASP types (OP1, OP3 and OP6), suggesting mechanisms that regulate pregnancy-induced responses in all major mammary epithelial cell types [16, 18, 28, 34, 106] (Fig. 3F).

Overall, our approach to profile molecular mechanisms regulating cellular states and pregnancy-induced development in organoid systems provided a solid framework for the utilization of such approached to further expand our understanding of master regulators of mammary lineage identity and development.

### Defining the Molecular Alterations Induced by Pregnancy Hormones on Human Mec-Derived Organoids

#### Characterizing the Developmental Timeline of Human Mammary Organoids Treated with Pregnancy Hormones

The current understanding of tissue alterations in response to pregnancy signals is largely biased towards the investigation of molecular and cellular dynamics in rodent models. Given that normal, human breast tissue has been utilized for the development of organoid systems [9, 35, 97, 102], we next decided to test their response to supplementation with EPP.

In doing so, we utilized an already established and characterized normal breast organoid culture, generated from breast specimens from women undergoing cosmetic reduction mammoplasty [9]. Human organoid cultures were treated with the same concentration of estrogen, progesterone and prolactin that was employed for mouse mammary organoids, given that human MECs have been shown to engage on pregnancy-induced development in response to pregnancy in mice [56]. Pregnancy-induced development was confirmed with the quantification of CSN2 mRNA levels, previously described to increase in response to pregnancy hormones [72, 96]. qPCR analysis indicated significant increased levels of pregnancy-specific CSN2 mRNA in contrast to CSN3 levels, starting on day 10 after EPP treatment, a response that was sustained up to 21 days of culturing (Fig. S4A). This observation was confirmed by the detection of CSN2 protein in human organoid cultures treated with EPP for 21 days (Fig. S4B). Therefore, we utilized the same culturing conditions for the generation of scRNAseq profiles of untreated and pregnancy-hormone treated human mammary organoids.

#### Cellular Identities of Human Mammary Organoids Treated with Pregnancy Hormones

Our analysis identified 9 clusters of human organoids (referred thereafter as HOP) (Fig. S4C). Characterization of lineage identity, utilizing classic markers of luminal and basal breast epithelial cells, indicated that the majority of cells in untreated and EPP-treated organoid cultures bear both luminal and basal traits, defined by the expression of *KRT8*, *KRT18*, *KRT5* and *KRT14*, suggesting that independent of treatment, established human breast organoid system have a more generalized mix-lineage signature (Fig. 4A). This observation agrees with previous studies describing that human breast organoid systems assume a more basal-like cellular phenotype after several culture passages, with consecutive loss of hormone receptor expression [9]. Interestingly, analysis of *KRT14* mRNA levels indicated clusters with high, low, and moderated levels of expression, suggesting that at least 3 epithelial lineages could be delineated (Fig. S4C). Therefore, and with the goal to define cellular states of established human breast organoid cultures, we employed an approach that utilized top differentially expressed genes across all clusters, and markers previously utilized to define human MECs identities [42] (Fig. 4B-C).

With this approach, we identified a unique population of LHS progenitor cells (HOP3), present in all culturing conditions, which expressed low levels of *KRT14*, and were defined by the expression of progenitor markers *FDCSP* and *ODAM* [52, 73], pregnancy hormone regulated genes (*BIRC3*) [57], and proliferating cell markers such as *MKI67, CCBN2* and *PTTG1* [81, 132] (Fig. 4B-C and S4C-D). Further analysis identified cell populations that were biased towards untreated human organoid samples, encompassing 2 populations of BMyo cell types (HOP2 and HOP7) which expressed high levels of *KRT14* mRNA, and high levels of basal-like cell identity such as *TTYH1, BT2, ISG15,* and *SH3KBP1* [12, 54, 105] (Fig. 4B-C and S4C and S4F). Interestingly, the expression of many of these genes were elevated across additional clusters, further supporting a more basal-like phenotype to human breast organoid cultures (Fig. 4B). We also identified a population of LASP cells (HOP4) to be more abundant in organoids without hormone treatment, and marked by the expression of lactogenic-associated genes such as *SLC2A1*, *NDRG1* and *EGLN3* [86, 133, 137] (Fig. 4B-C and S4F). Collectively, our analysis suggests the existence of population of cells that are present in human breast organoid conditions, and that are negatively impacted by the presence of pregnancy hormones.

We next focused on the characterization of cellular clusters biased to EPP-treated conditions. This approached identified cell types spanning a series of LHS states, mostly marked by lower levels of *KRT14* mRNA, and variable levels hormone responsive genes such as *BIRC3, RARRES1,* and *NUPR1* (HOP1, HOP3, HOP5, HOP6, HOP8 and HOP9) [10, 43, 81, 138] (Fig. 4B-C, and S4C and S4F). In addition, we defined populations of LASP cells expressing estrogen/progesterone-associated genes such as *AREG, ODAM,* and *FKBP5* [13, 39, 51] (cluster HOP1), and those expressing prolactin-genes such as *TSC22D3, NDRG1* and *VEGFA* [74, 92, 93, 114] (cluster HOP8), thus illustrating a degree of cell specificity in response to pregnancy hormones (Fig. 4B-C). Moreover, we also identified differentiated population of LHS cells, marked by the expression of *CLND3* (clusters HOP5 and HOP6), and differentiated LHS cell cluster HOP9, which was biased towards conditions treated with EPP for 10 days, marked by the expression of *FXYD3* and *LCN2* [66, 134] (Fig. 4B-C).

We also identified specific cytokeratin markers that further defined cellular states of human breast organoid cultures, with high levels of *KRT6* marking LASP and BMyo cells (clusters HOP1, HOP2, HOP4, and HOP8), and high levels of *KRT7* marking EPP-induced mature LHS cells (HOP5, HOP6, and HOP9) (Fig. S4E). Collectively, this analysis identified distinct cellular states, based on alterations to gene expression and organoid treatment response, thus illustrating the complex cellular dynamics induced by pregnancy hormones.

#### Signaling Pathways Active During Responses to Pregnancy Hormones in Human Mammary Organoids

We next employed a general gene expression analysis, to indicate potential pathways enriched in breast epithelial organoid cultures. In doing so, we first examined enriched pathways of each cellular cluster from untreated human breast organoids. While luminal clusters defined to have a high proliferative state were marked by pathways associated with cell cycle regulation (clusters HOP3, HOP5), proliferating BMyo cluster HOP7 was marked by pathways linked with interferon responses, signals know to regulate the growth dynamics of epithelial cells [24, 89], thus suggesting distinct mechanisms of cell growth regulation in organoid systems (Fig. S4G). Interestingly, BMyo2 cells show no enrichment for a particular pathway, further suggesting an overall up-regulation of basal-like programs across populations of human breast organoid cultures. Clusters of LHS cells were enriched with pathways associated with hormone response, such as TNF-⍺ signaling via NF-κB pathways (clusters HOP1 and HOP6), and mTOR signaling, (clusters HOP4, HOP6 and HOP8) [53, 78, 99] (Fig. S4G). Cluster HOP4 and HOP6 were also enriched for genes associated with Hypoxia, thus suggesting cellular states with increased metabolic rates (Fig. S4G).

A similar analysis approach was employed to define the transcriptional state of cellular clusters in organoid cultures grown EPP (Fig. S4H-J). Our results suggest that while hormone clusters HOP5 and HOP6 show no enrichment for pathways when compared with no treatment, a metabolic state switch is suggested with prolonged pregnancy hormone exposure (21 days), with cluster HOP5 downregulating fatty acid associated signaling and HOP6 up-regulating process linked with p53 pathway, hypoxia and mTORC1 (Fig. S4H-J). Hypoxic-associated pathways were also identified in clusters HOP1 after 10 days of EPP culturing, and in clusters HOP8 and HOP9 across both EPP-treated conditions, further supporting the effects of pregnancy hormones on regulating the metabolic state of breast organoid cells (Fig. S4H-J).

Interestingly, cells from cluster HOP9 were enriched for pathways associated with EMT and c-MYC targets in response to prolonged exposure to EPP (21 days), thus suggesting the activation of cell plasticity process associated with pregnancy signals (Fig. S4J). Collectively, these findings illustrate the molecular and cellular alterations, induced by ex vivo exposure to pregnancy hormones, thus supporting the robustness of organoid cultures to understand normal developmental stages of human breast tissue.

#### Cellular Identities of Integrated Murine and Human Organoids Without Hormone Supplementation

Our analysis indicated that both murine and human mammary organoids treated with EPP recapitulated some of the previously described pregnancy-induced changes that take place in vivo. Yes, it is possible that pregnancy signals may activate pathways that are both evolutionary conserved and species specific. Therefore, we set out to define the evolutionary conserved basis of mammary organoid systems between human and murine cultures, by initially integrating untreated murine and human organoids datasets (referred hereafter as UMH clusters). Such approach identified a total of 7 clusters with varied distribution across species (Fig. S5A). To avoid lineage classification issues, biased by the state of human organoid cultures, we utilized once again the top differentially expressed genes to determine the identities of each UMH cluster (Fig. S5B).

Our analysis identified five clusters of luminal-biased cell types (UMH1, UMH3, UMH4, UMH5, and UMH7), from each two clusters were classified as LASP state (UMH1 and UMH4), and three clusters defined to be of LHS lineage (UMH3, UMH5, and UMH7), including two defined to be at high proliferative state (UMH3 and UMHM5). We also identified 2 clusters of BMyo cell types (UMH2, and UMH6), thus further supporting the heterogeneity of organoid derived from mammary tissue (Fig. S5B-D).

The distribution of organoid clusters also varied according to species. While clusters UHM1 (LASP) and UMH6 (BMyo) were biased towards samples from murine origin, BMyo (UMH2) and LASP (UMH4) fates were also identified in human organoid cultures, thus suggesting a species-specific distribution of these lineages in organoid cultures (Fig. S5A-D). Our analysis also identified clusters of cell populations somewhat present in both mouse and human organoid conditions, mostly represented by LHS lineages, including those at a high proliferating state (Fig. S5A-D). The aforementioned observations concur with previous findings comparing intact human and murine MECs, where luminal lineages, especially progenitor-like ones, were shared across species [42]. Collectively, this approach allowed for the initial identification of species biased organoid cell types.

#### Cellular Identities of Integrated Murine and Human Organoids with Hormone Supplementation

We next asked whether treatment with pregnancy hormones would influence the dynamics of species-specific mammary epithelial subtypes. In doing so, we integrated EPP-treated murine and human organoids datasets (referred hereafter as PMH clusters), an approach that yield 9 cellular clusters of several epithelial lineages (Fig. 4D-F, and S5E). Our analysis once again identified cell populations that are shared across species, and those that are species specific, thus illustrating differences to how breast organoid cultures from mouse and human mammary glands respond to pregnancy hormones (Fig. 4D-F).

For example, BMyo cell types (PMH7) and BL cell types (PMH9) were identified to be biased to mouse organoids treated with EPP, perhaps illustrating distinct alterations to cellular states in response to pregnancy hormones compared to human organoids (Fig. 4D-F). In addition, we identified several clusters of cells bearing high levels of *CSN3* mRNA, a gene that is associated with a LASP state in clusters that were either made up of mouse and human MECs (cluster PMH1) or in clusters biased towards mouse organoid conditions that also expressed elevated *PRLR* mRNA levels (clusters PMH3, PMH8 and PHM9) (Fig. 4D-F, and S5E). These observations suggest that prolactin-induced responses are more efficiently activated in murine systems supplemented with EPP.

Human organoid biased clusters were identified to bear an LHS state (cluster PMH5), a cell type showed to be expanded by EPP, thus indicating selective responses by organoid treatment (Fig. 4D-F). In fact, additional clusters of cells with representation in both murine and human datasets were classified to have an LHS lineage, including those in highly proliferative states, that were induced by EPP treatment, (Fig. 4E-F). Therefore, our findings support that our organoid conditions, to some extent, recapitulates pregnancy-induced development observed in both mouse and human mammary systems.

## Discussion

Our characterization of MEC-derived organoids at a single-cell level allowed us to carry out a comprehensive assessment of organoid systems to model mammary gland development. Our initial analysis of murine MEC-derived organoids scRNA-seq data confirmed conservation of in vivo lineage signatures, as well as representation of a diverse array of MEC lineages ex vivo. These results complement a previous proteomics study that made use of Cytometry by time of flight (CyTOF) to confirm that MEC lineages found in vivo are present in patient MEC-derived organoid cultures [35]. We further confirmed lineage fidelity between in vivo and 3D ex vivo systems by comparing scRNA-seq data from intact murine mammary tissue to data we generated from murine MEC-derived organoids. This particular analysis resulted in the appearance of a luminal progenitor population that is organoid exclusive, suggesting that certain cells in culture exist in a stem-like state, potentially to maintain the growth of cells ex vivo. Therefore, our results demonstrate the fidelity and discrepancies between in vivo and ex vivo mammary tissue systems.

The induction of MECs into an immature cellular state in organoid cultures could have resulted from a lack of microenvironment queues that are crucial for mammary development. For example, prior research has highlighted the significance of various fibroblast types in MEC development and homeostasis, as well as the potential role of adipocytes in regulating MEC growth and function stages [36, 44, 45, 63, 70, 131]. Moreover, signals that can result from paracrine signaling from other tissues are also vital for the maturation of specific MECs, such as oxytocin, which promotes the differentiation of myoepithelial cells [104]. Medium composition has also been shown to affect organoid culture composition [35], which could also have contributed to the observed phenotype. Nonetheless, analysis of regulons specific to each system highlighted their role in supporting survival and achieving homeostasis within their respective microenvironments. Thus, MEC-derived organoids are a suitable system to assess the effect of controlled developmental signals, but should be used with the previously discussed considerations. Future studies involving the addition of signals that contribute to endogenous mammary gland development and maintenance, along with co-culturing with essential cells from the mammary microenvironment will further improve the fidelity of organoid systems [55, 119, 121].

Single-cell characterization of murine MEC-derived organoids treated with different concentrations of estrogen enabled us to begin to isolate the effects of individual hormones on MEC development, especially during distinct biological processes involving an interplay of varying hormone doses (e.g. the estrus cycle). This analysis revealed the emergence of an estrogen-exclusive LHS population, as well as a depletion of mixed lineage cells exclusively at a high dose of estrogen (66.6 ng/mL). Our results suggest that LHS cells in our estrogen-exclusive cluster are not an emerging cell type, but rather a cell state triggered by hormone supplementation. This is evidenced by the simultaneous depletion of a cellular cluster of LHS cells that is enriched in untreated samples. Further comparison of both LHS clusters revealed that estrogen-exclusive LHS cells highly express *Areg* and *Pgr*, both which have been previously described to be upregulated by estrogen [50]. Moreover, our findings that estrogen-exclusive LHS cells are highly differentiated compared to untreated LHS cells indicate that hormone treatment could be promoting cellular maturation, in accordance with previous studies [4]. These mature LHS cells also displayed an activation of pathways associated with proliferation and inflammation, which have been previously linked to estrogen-mediated activation [69].

A lack of hormone signals at baseline could further explain why we observe an enrichment of mixed lineage cells in organoids without treatment and a stark depletion in organoids treated with a high dose of estrogen. This interpretation is complementary to a previous study that delineates a quiescent state for mixed lineage cells in the adult mammary gland, which become active in the presence of hormones [32]. Therefore, these results highlight that organoid culturing conditions at baseline resemble developmental stages depleted of hormones, such as prepubescent development and menopause. Given that an aged extracellular matrix alone can drive MECs into neoplastic and invasive cellular states [5], it will be important to identify what stages of development the composition of Matrigel and organoid media resembles most. Thus, our analysis paves the way to future studies that will involve comparing organoid MECs with intact MECs from pre-pubescent and post-menopausal mice.

Previous work using a combination of prolactin, hydrocortisone, OT, and growth factors showed mouse MEC-derived organoids are able to mimic lactation and involution [120]. Additional studies further introduced the idea of using a cocktail of pregnancy hormones (estrogen, progesterone and prolactin, or EPP) to simulate a pseudo-lactation state, which resulted in the incremental expression of *Csn2* and changes to the epigenome previously associated with pregnancy [21]. Our current study extends upon these studies by demonstrating compositional and transcriptomic changes to mammary organoids as a direct effect of treatment with pregnancy hormones. We show a depletion and emergence of similar cell types with pregnancy hormones treatment, suggesting that the observed compositional changes in organoids with pregnancy hormones are likely due to subtle changes in cellular states. Moreover, cellular clusters that emerge with pregnancy hormones treatment are enriched for processes that have been previously associated with lactation, such as adipogenesis and hypoxia [22, 108]. Therefore, these results indicate specific cell types obtain a parity-associated gene expression signature with exposure to hormones during pregnancy. We further compared scRNA-seq data from MECs obtained at intact pregnancy stages [4] with our organoids treated with pregnancy hormones, and found our organoid cultures recapitulate lineages from all pregnancy stages.

We also found that our organoids possess a cellular state that is only found in MECs undergoing gestation, thus suggesting that the proliferative and stem-like state of organoid MECs is most similar to this stage of pregnancy. Therefore, we conclude that organoids can recapitulate drastic cellular changes that occur with pregnancy, particularly by mimicking the gene signature of MECs during pregnancy. However, since organoid MECs at baseline appear to have additional levels of proliferation than nulliparous MECs, this model must be used with caution to understand pregnancy-associated development. In fact, analysis comparing untreated and treated organoid cultures identified a population of LHS MECs largely exclusive to conditions supplemented with pregnancy hormones, thus supporting a possible cellular expansion in response to pregnancy signals (Fig. 3A-B). Interestingly, the existence of pregnancy-induced MECs (PI-MECs) has already been suggested in intact mammary tissue, although its true lineage identity and function remain very controversial [17].

We were able to uncover the translational potential of MEC-derived organoids by further showing that patient MEC-derived organoids respond to pregnancy hormones by inducing transcriptomic changes to organoid MECs associated with pregnancy. Interestingly, similar to our observation in murine organoids, one of the enriched pathways in human organoids treated with pregnancy hormones were those associated with hypoxia, reflecting its significance in pregnancy where the mammary gland boosts metabolic activity to support growth and lactogenesis, thereby activating hypoxia-associated genes [108].

Nonetheless, we found that most human organoid MECs exist in a luminal-basal state. The phenomenon of organoids becoming more basal-like after long term culturing had already previously been reported [9], thus potentially confirming that the phenotype we observed in human organoids could be a result of the number of passages prior and during the course of the experiment. One approach that could be implemented to address this issue is to grow the cells and sequence them right before the next passaging, allowing cells to differentiate in culture prior to sequencing. However, there are other factors that could affect the observed phenotypes in culture, such as the inability to remove growth factors from culture due to the developmental timeline of human organoid MECs compared to murine organoids. Notably, despite the mixed lineage phenotype we observed, we did identify LHS cells with low levels of ERα expression and expression of downstream estrogen targets in these human MEC-derived organoid cultures. It has long been a challenge to obtain hormone positive clones in culture, as previous studies using BC-derived organoids have noted that the expression of hormone receptors is reduced in culture compared to intact tissue [15, 37]. Our identification of LHS clones in normal MEC-derived organoids using both a combination of hormone receptor status and downstream targets therefore suggests the potential of using these 3D cultures for understanding the development of hormone positive BCs, extending the applications of 3D cultures towards both fundamental biological research and potential clinical implications.

When comparing murine and human organoids, mature cells clustered mainly in a species-specific manner, preserving the suggested hierarchy across species while displaying divergent epithelial responses, as it has been reported in previous literature [42]. Interestingly, a subset of proliferative LHS cells was identified in both human and murine MEC organoid cultures, suggesting a conserved population that plays a crucial role in maintaining mammary tissue homeostasis throughout evolution. However, upon treatment with pregnancy hormones, further significant differences in mature cell types emerged between the species. The compositional differences observed in milk from various mammalian species, influenced largely by phylogeny, imply intrinsic cellular response variations [111]. Notably, the shared LHS cell population between mice and humans in cultures treated with pregnancy hormones appeared to be divided between proliferating and non-proliferating cells, indicating potential expansion and distinct functions in preparing the mammary gland for lactation. Moreover, shared cluster signatures across species in response to pregnancy hormones highlighted processes associated with pregnancy, such as hypoxia, estrogen response, and fatty acid metabolism [22, 108]. Therefore, our findings shed light on the intricate interplay between species-specific and conserved cellular responses in the context of mammary tissue dynamics and lactation preparation.

Altogether, we have developed an atlas of normal MEC-derived organoids from mouse and human tissue, which can be incorporated with other single-cell methods to understand the molecular mechanisms governing MEC development ex vivo. We characterize the effects of feminizing hormones on these 3D cultures at a single-cell level, supporting hormone treatment of organoids as a system to understand developmental processes associated with adolescence, pregnancy and menopause. Our findings support the implementation of this procedure as a non-invasive method to understand how the human mammary gland is modified during a pregnancy cycle. This system can also be extended to other species, in order to assess the evolutionary basis of MEC response to hormones across other mammalian species.

### Supplementary Information

**Additional file 1.**

**Additional file 2.**

## Data Availability

scRNA-seq datasets were deposited into NCBI database [https://www.ncbi.nlm.nih.gov/], BioProject PRJNA1015687, and will be made available upon manuscript acceptance/publication. scRNA-seq datasets will also be uploaded into the web platform CZ CELLxGENE (CZI Initiative), and our lab GitHub (https://github.com/dosSantosLabCSHL?tab=repositories), for user friendly data access/analysis, upon manuscript publication. Previously published datasets are available under the following IDs: SAMN16776241 (re-clustered MECs from nulliparous BALB/c mice), GSM2834498, GSM2834499 (mammary tissue from nulliparous C57BL/6 female mice), GSM2834500, GSM2834501 (mammary tissue from C57BL/6 female mice at mid-gestation), GSM2834502, GSM2834503 (mammary tissue from C57BL/6 female mice during lactation), GSM2834504, GSM2834505 (mammary tissue from nulliparous C57BL/6 female mice at late involution stage).
