# Supplementary material for: Single-Cell Transcription Mapping of Murine and Human Mammary Organoids Responses to Female Hormones
Source: J Mammary Gland Biol Neoplasia. 2024 Jan 30;29(1):3. doi: 10.1007/s10911-023-09553-x (PMC10827859; doi:10.1007/s10911-023-09553-x)
Supplement: Supplementary file 1 — Additional file 1. [file 10911_2023_9553_MOESM1_ESM.docx]

**Supplementary Figures**

**Supplementary Figure S1. Approaches for MO and OIM characterizations.** (A) Dotplot showing MEC markers average gene expression in each MO cluster. (B) GSEA for enriched hallmark terms in cluster MO3 vs all other clusters. The terms are ordered based on each –log(nom p-val) for each term. Resulting terms were not filtered for significance given that none passed the p-val < 0.05 threshold. (C) Dotplot for MEC marker expression from OIM clusters. OIM clusters are organized based on dendrogram relationships.

**Supplementary Figure S2. Strategies for classifying OE clusters.** (A) Resulting OE clusters with identities based on intact MEC marker expression. (B) Dotplot for OE clusters expression of intact MEC gene markers. OE clusters are organized based on dendrogram relationships. (C) Cell cycle scoring or OE clusters.

**Supplementary Figure S3. Classification strategies for OP and OIP clusters.** (A) Resulting clusters of murine organoids with and without EPP (OP), with their identifications corresponding to expression of previously known MEC markers. (B) Dotplot for MEC marker expression in OP clusters. Clusters are organized based on dendrogram relationships. (C) Cell cycle scoring of OP clusters. (D) GSEA for hallmark terms enriched in each OP cluster. Terms are ordered from highest –log(nom p-value). Only hallmark terms with a normalized p-val < 0.05 were kept for this analysis. The color of each dot represents the NES for each term. The red boxes mark clusters depleted with EPP treatment, and the purple boxes mark clusters enriched with EPP. (E) Dotplot for MEC marker expression in OEP clusters. Clusters are organized based on dendrogram relationships. (F) Module score analysis for markers from cluster OE6 in OP clusters. The blue arrows point to clusters with a high score for OE6 markers, clusters OP2, OP4, OP7, OP8, OP9 and OP10. (G) Resulting clusters of murine organoids with and without EPP treatment integrated with data sets from intact pregnancy MECs (OIP), with their identifications corresponding to expression of previously known MEC markers. (H) Dotplot for MEC marker expression in OIP clusters. Clusters are organized based on dendrogram relationships. (I) Bar plot showing percentage of cells per condition in each OIP cluster. The blue arrow highlights cluster OIP7, which is enriched in EPP-treated organoids. (J) Cell cycle scoring of OIP clusters.

**Supplementary Figure S4. Strategies for determining the optimal timeline and treatment effects of EPP on human organoids through gene expression analysis.** (A) qPCR results for CSN2/CSN3 expression in human MEC-derived organoids treated with EPP for 21 days (n=2 runs per day). Comparing CSN2 expression at 0 days and 21 days of EPP treatment resulted in a significant difference in expression (p-val = 0.0238). (B) Immunofluorescence stain for milk-associated protein CSN2 in human organoids treated with pregnancy hormones (EPP). (C) Violin Plots showing the KRT14 levels of expression (high, moderate or low) used to classify luminal and basal populations within each HOP cluster, divided by condition (no EPP treatment, early EPP treatment or D10 and late EPP treatment or D21). (D) Cell cycle scoring of each HOP cluster. (E) Violin Plots showing the expression of additional cytokeratins used to classify luminal and basal populations within each HOP cluster. (F) Bar plot showing percentage of cells per condition in each HOP cluster. The red arrow highlights HOP2, most notably enriched in EPP treatment at 10 and 21 days. The blue arrow highlights HOP9, which is depleted during late (21 days) EPP treatment. (G) GSEA for enriched hallmark terms in each HOP cluster with no EPP treatment. Terms are ordered based on–log(nom p-value). Only hallmark terms with a normalized p-val < 0.05 were kept for this analysis. The color of the dots represents NES. (H-J) GSEA for hallmark terms differentially enriched in EPP-time point across clusters. Terms were ordered decreasingly based on –log(nom p-value). Only terms with nom p-val <0.05 were kept for these analyses. Likewise, the color of the dots represents NES.

**Supplementary Figure S5. Additional transcriptomic analyses for PMH clusters.** (A) Integration of data sets from murine and human organoid MECs without treatment (Untreated and Human MEC organoids – UHM). The blue arrow highlights a cellular cluster shared between species. (B) Dotplot showing expression of the top DEGs per UHM cluster. (C) Cell cycle scoring of UHM clusters. (D) Bar plot showing percentage of cells per condition in each UHM cluster. Clusters enriched in both humans and mice are highlighted by the blue arrow. (E) Resulting PMH clusters with their respective cluster identities. (F) Cell cycle scoring of PHM clusters.
